# *Nod2* protects mice from inflammation and obesity-dependent liver cancer

**DOI:** 10.1038/s41598-020-77463-7

**Published:** 2020-11-25

**Authors:** Serdar A. Gurses, Sunil Banskar, Cody Stewart, Bill Trimoski, Roman Dziarski, Dipika Gupta

**Affiliations:** Indiana University School of Medicine–Northwest, Gary, IN 46408 USA

**Keywords:** Cancer models, Tumour immunology

## Abstract

Nod2 is a pattern recognition receptor that modulates host innate immune responses and protects from inflammation, steatosis, and obesity. Obesity and inflammation are risk factors for hepatocellular carcinoma, however, the role of Nod2 in obesity-dependent hepatic tumorigenesis is not known. Here we tested the hypothesis that *Nod2* protects from high fat diet (HFD)-dependent hepatic cancer. We used an obesity-dependent hepatic tumor model. WT and *Nod2*^−/−^ mice were treated with the carcinogen dimethylbenz[a]anthracene (DMBA) and maintained on HFD. *Nod2*^−/−^ mice treated with DMBA and maintained on HFD gain significantly more weight and develop more liver tumors than similarly treated WT mice. Livers of *Nod2*^−/−^ tumorigenic mice had increased expression of genes involved in cell proliferation, immune responses, and cholesterol biosynthesis, increased infiltration of neutrophils, inflammatory monocytes, and T cells, and increased activation of STAT3 and ERK during the later stages of tumorigenesis. Bioinformatic analyses of genes with differential expression predicted an increase in cancer, immune, and cholesterol biosynthesis pathways. In summary, we have identified a novel role for *Nod2* and demonstrate that *Nod2* protects from HFD-dependent liver malignancy and this protection is accompanied by decreased cell proliferation, inflammation, steroid biosynthesis, neutrophils and macrophages infiltration, and STAT3 and MAPK signaling in the liver.

## Introduction

Liver cancer is the fifth most frequent malignancy and one of the leading causes of cancer-related deaths worldwide. There are approximately 700,000 deaths worldwide each year, and the 5-year survival rate in patients with liver cancer is a dismaying 18%^1–3^. Obesity is a significant risk factor for liver cancer, and the rising epidemic in obesity parallels increasing incidence in hepatic malignancy^1,2^. The development of liver cancer in obese individuals is accompanied by chronic inflammation, which implicates a role for the immune system in hepatic tumorigenesis in these individuals^4–6^. However, specific host immune factors and molecular mechanisms that contribute to the development of liver cancer remain poorly understood^5–7^.

The Nod-like receptors (NLRs) are intracellular innate immunity proteins. Nod2, one of the members of the NLR family, is a sensor for bacterial peptidoglycan fragments and has a critical role in innate immune responses to bacteria^8–10^. Activation of Nod2 by peptidoglycan results in the activation of NF-κB and MAP kinase-signaling cascades and production of inflammatory molecules and anti-microbial peptides^8–12^. In addition to bacterial peptidoglycan, there is increasing evidence that Nod2 is likely activated by other ligands, including cellular ligands released during stress^13–16^. In vivo, *Nod2* has a critical role in maintaining intestinal homeostasis and is a major susceptibility gene for inflammatory bowel disease^17,18^. Inflammation is a risk factor for some cancers, and polymorphisms in *Nod2* are associated with increased risk for colorectal cancer^19–21^ and may be associated with increased risk for lymphoma, gastric, breast, ovarian, lung, and laryngeal cancers^21^. In experimental models, *Nod2* deficiency was shown to promote the development of colitis and colorectal cancer^22,23^. *Nod2*-dependent protection from colorectal cancer was dependent on the inhibition of TLR-mediated activation of NF-κB and MAPK signaling^23^. Recently, *Nod2* was shown to protect from the development of hepatocellular carcinoma (HCC) using N-nitrosodiethylamine (DEN)/carbon tetrachloride (CCl_4_) and xenograft tumor animal models^24^. In vitro, molecular changes associated with HCC were dependent on the *Nod2*-dependent activation of adenosine 5′-monophosphate activated protein kinase (AMPK) and increased apoptosis^24^. Dysregulation of *Nod2* is also linked to metabolic and endoplasmic reticulum stress^13–15,25^. However, the functional role of *Nod2* in these different pathologies remains poorly understood.

We previously demonstrated that *Nod2* protects from the development of diet-dependent obesity^15^. *Nod2*^−/−^ mice on high fat diet (HFD) become obese, develop steatosis, and exhibit increased expression of genes for a large number of inflammatory molecules, compared with wild-type (WT) mice on HFD^15^. We also demonstrated that the *Nod2*^−/−^ HFD microbiome contributes to the development of obesity and steatosis in these mice^15^. However, the role of *Nod2* in the development of diet-dependent hepatic malignancy is unknown. In the current study, we tested the hypothesis that increased obesity in the absence of *Nod2* would result in increased tumorigenesis. We used the dimethylbenz[a]anthracene (DMBA)-HFD mouse model, which is an established system for the development of obesity-dependent liver tumors^26^. We show that *Nod2* inhibits hepatic tumorigenesis and that the development of liver tumors in *Nod2*^−/−^ mice is dependent on HFD and involves increased expression of genes involved in cell proliferation, immune responses, and cholesterol biosynthesis. We further demonstrate that the development of liver tumors in *Nod2*^−/−^ mice is associated with increased infiltration of neutrophils, inflammatory monocytes, and T cells in the liver, and with increased activation of STAT3 and ERK signaling molecules.

## Results

### *Nod2* protects from obesity-dependent liver cancer

To test our hypothesis that development of diet-dependent obesity in *Nod2*^−/−^ mice increases sensitivity to liver cancer, we treated WT and *Nod2*^−/−^ 4–5 day old pups one-time with the carcinogen dimethylbenz[a]anthracene (DMBA) and 3 days later placed their nursing mothers on HFD. The DMBA-treated mice were continued on HFD after they were weaned and until the end of the experiment. This is an established method for the development of obesity-induced liver cancer in mice^26^. DMBA causes an oncogenic Ras mutation affecting a pathway that is frequently activated in human cancers, but by itself is not sufficient to cause liver cancer in this mouse model^26^. Control WT and *Nod2*^−/−^ mice were not treated with DMBA, but were started and maintained on HFD similar to the DMBA + HFD group or were treated with DMBA and maintained on chow.

**Figure 1:**
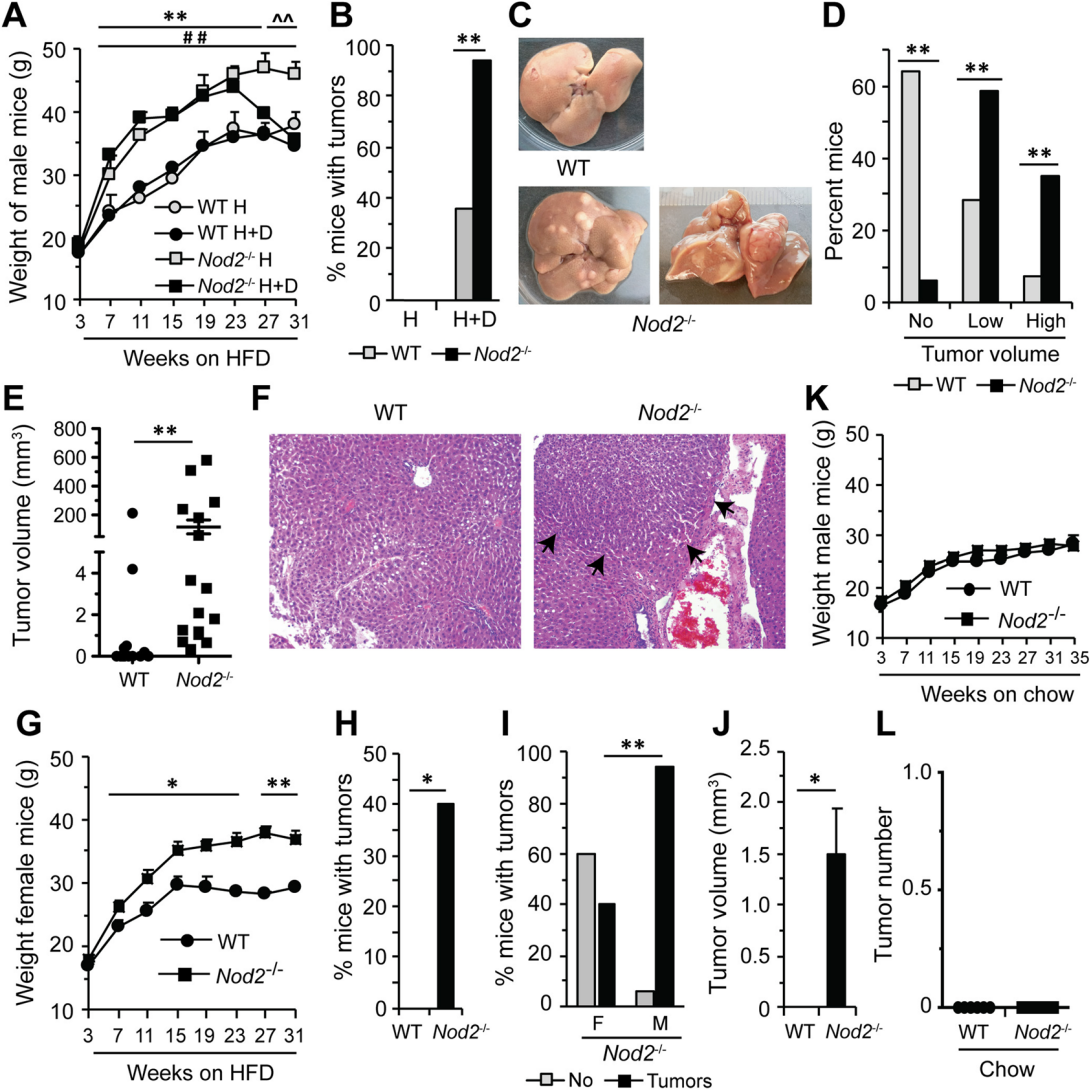
*Nod2*^−/−^ DMBA + HFD mice become obese and develop liver tumors. WT and *Nod2*^−/−^ 4–5 day old pups were treated with a single dose of DMBA, maintained on (**A**–**J**) HFD (H + D), or (**K**, **L**) chow, or (**A**, **B**) not treated with DMBA and maintained on HFD (H) for the length of the experiment. All mice were monitored for weight gain starting at the time of weaning and weighed every other week for the length of the experiment and were monitored for the development of liver tumors after 31 weeks. (**A**–**F**) Male mice, (**A**) mean body weight ± SEM, (**B**) percent mice with tumors, WT H *N* = 0 out of 6, *Nod2*^−/−^ H *N* = 0 out of 6, WT H + D *N* = 5 out of 14 and *Nod2*^−/−^ H + D *N* = 16 out of 17, (**C**) representative images of liver showing tumors, (**D**) percent mice with no tumors or small or large tumors, (**E**) tumor volume in individual mice, and (**F**) representative hematoxylin and eosin stained liver sections; arrows indicate the boundary of the tumor mass. (**G**, **H**, **J**) Female mice, (**G**) mean body weight of ± SEM. (**H**) percent mice with tumors, WT *N* = 0 out of 10 and *Nod2*^−/−^ *N* = 4 out of 10, (**I**) percent mice with tumors comparing male (M) and female (F) *Nod2*^−/−^ mice, and (**J**) mean tumor volume ± SEM. (**K**) mean body weight ± SEM and (**L**) number of tumors in WT and *Nod2*^−/−^ male mice on chow. *N* = 6–15 mice/group. Significance of difference (**A**, **E**, **G**, **J**, **K**, **L**) by *t-test* and (**B**, **D**, **H**, **I**) by Chi square. *Nod2*^−/−^ H + D *versus* WT H + D, *P ≤ 0.05, **P ≤ 0.01, *Nod2*^−/−^ H *versus* WT H, ^##^*P* ≤ 0.01, and *Nod2*^−/−^ H + D *versus Nod2*^−/−^ H, ^^^^*P* ≤ 0.01.

We observed a dramatic increase in weight gain by male *Nod2*^−/−^ mice compared with male WT mice in both HFD and HFD + DMBA groups (Fig. 1A). For both *Nod2*^−/−^ groups there were significant differences in weight starting from week 7 and *Nod2*^−/−^ HFD mice continued to gain weight up to 31 weeks (last time point). However, the *Nod2*^−/−^ HFD + DMBA mice started losing weight after 23 weeks, and at 27 and 31 weeks there was a significant difference between *Nod2*^−/−^ HFD and *Nod2*^−/−^ HFD + DMBA mice. The loss in weight in *Nod2*^−/−^ HFD + DMBA mice at these later time points is likely due to the development of tumors in these mice, which is described below. At 31 weeks, 94% of male *Nod2*^−/−^ HFD + DMBA mice had liver tumors, whereas only 36% of male WT HFD + DMBA mice developed liver tumors (Fig. 1B). WT HFD and *Nod2*^−/−^ HFD male mice did not develop any tumors (Fig. 1B). The majority of tumors in WT HFD + DMBA mice were small (low volume, 0.2 to 4 mm^3^) and only one mouse had a large tumor (high volume, 50 to 600 mm^3^). In contrast, *Nod2*^−/−^ HFD + DMBA mice had significantly higher numbers of both low and high-volume tumors compared with WT mice (Fig. 1C,D,E). A large tumor mass with small undifferentiated cells in *Nod2*^−/−^ HFD + DMBA liver is shown in Fig. 1F.

Female *Nod2*^−/−^ mice treated with DMBA and maintained on HFD gained significantly more HFD-dependent weight compared with similarly treated female WT mice (Fig. 1G). This increased weight gain in *Nod2*^−/−^ mice is similar to our previous results with mice maintained on HFD but not treated with DMBA^15^. WT female mice did not develop any liver tumors whereas 40% of *Nod2*^−/−^ female mice developed liver tumors (Fig. 1H), which was significantly higher than WT female mice (Fig. 1H), but lower than male *Nod2*^−/−^ mice (Fig. 1I). The tumors in female mice were mostly small (low volume, 0.2 to 4 mm^3^, Fig. 1J). WT and *Nod2*^−/−^ mice treated with DMBA and maintained on chow gained similar weight (Fig. 1K) and developed no tumors (Fig. 1L). We had previously shown that female WT and *Nod2*^−/−^ mice maintained on HFD but not treated with DMBA do not develop tumors^15^.

Thus, our data indicate that *Nod2* plays a protective role in the development of diet-dependent hepatic malignancy. Male mice are more susceptible than female mice, however, both *Nod2*^−/−^ male and female mice are more susceptible than WT male and female mice, respectively. We further show that the development of hepatic tumors in *Nod2*^−/−^ mice is also dependent on both HFD and DMBA as mice treated with DMBA and maintained on chow or mice on HFD but not treated with DMBA (ref. 15 and current data) did not develop liver tumors.

### Development of obesity-dependent liver tumors in *Nod2*^−/−^ mice is associated with differential expression of genes in cancer, inflammatory, hepatotoxicity, and cholesterol biosynthesis pathways

To unravel the molecular mechanism underlying the development of obesity-dependent liver tumors in *Nod2*^−/−^ mice in a unbiased manner, we performed RNAseq using total RNA from the livers of male WT and *Nod2*^−/−^ mice treated with DMBA and maintained on HFD for 31 weeks. We focused on male mice because males are more susceptible to the development of liver tumors compared with female mice (Fig. 1I). *Nod2*^−/−^ mice had significantly increased expression of more than 600 genes and decreased expression of more than 400 genes with a 5% FDR (*P* ≤ 0.05) (Supplementary Fig. S1). We analyzed the data using Ingenuity Pathway Analysis (IPA) software (Qiagen) to identify Gene Ontology (GO) pathways and diseases that are differentially expressed between WT and *Nod2*^−/−^ mice. Based on the number of genes, the top three disease pathways that were significantly enhanced in *Nod2*^−/−^ mice were for cancer, inflammatory response, and immunological disease (Table 1). We further analyzed the data for genes associated with hepatotoxicity and show a significant enrichment of pathways associated with liver hyperplasia/hyperproliferation, steatosis, hepatocellular carcinoma, liver necrosis/cell death, and liver inflammation/hepatitis (Table 1). Amongst metabolic pathways, genes involved in steroid/cholesterol biosynthesis were significantly enriched in *Nod2*^−/−^ mice. Thus, our data demonstrate that the innate immunity gene *Nod2* protects mice from the altered expression of genes associated with the development of cancer, liver hyperplasia, liver carcinoma, and inflammation in the DMBA + HFD model of liver cancer.

**Table 1:** GO pathways that are differentially expressed in the liver of male *Nod2*^−/−^ DMBA + HFD mice compared with male WT DMBA + HFD mice.

	# genes	*P*-value
**Disease**		
Cancer	863	1.18E−04 to 1.96E−15
Inflammatory response	276	1.15E−04 to 5.50E−13
Immunological disease	288	1.17E−04 to 1.92E−12
**Hepatotoxicity**		
Liver hyperplasia/hyperproliferation	574	5.65E−01 to 1.88E−07
Liver steatosis	39	1.02E−01 to 1.86E−06
Hepatocellular carcinoma	71	3.94E−01 to 9.53E−04
Liver necrosis/cell death	24	6.08E−01 to 3.39E−03
Liver inflammation/hepatitis	30	1.00E00 to 5.53E−03
**Metabolism**		
Cholesterol biosynthesis	14	1.85E−12

### Development of obesity-dependent liver tumors in *Nod2*^−/−^ mice is associated with increased expression of genes involved in cell proliferation, immune responses, and lipid metabolism

**Figure 2:**
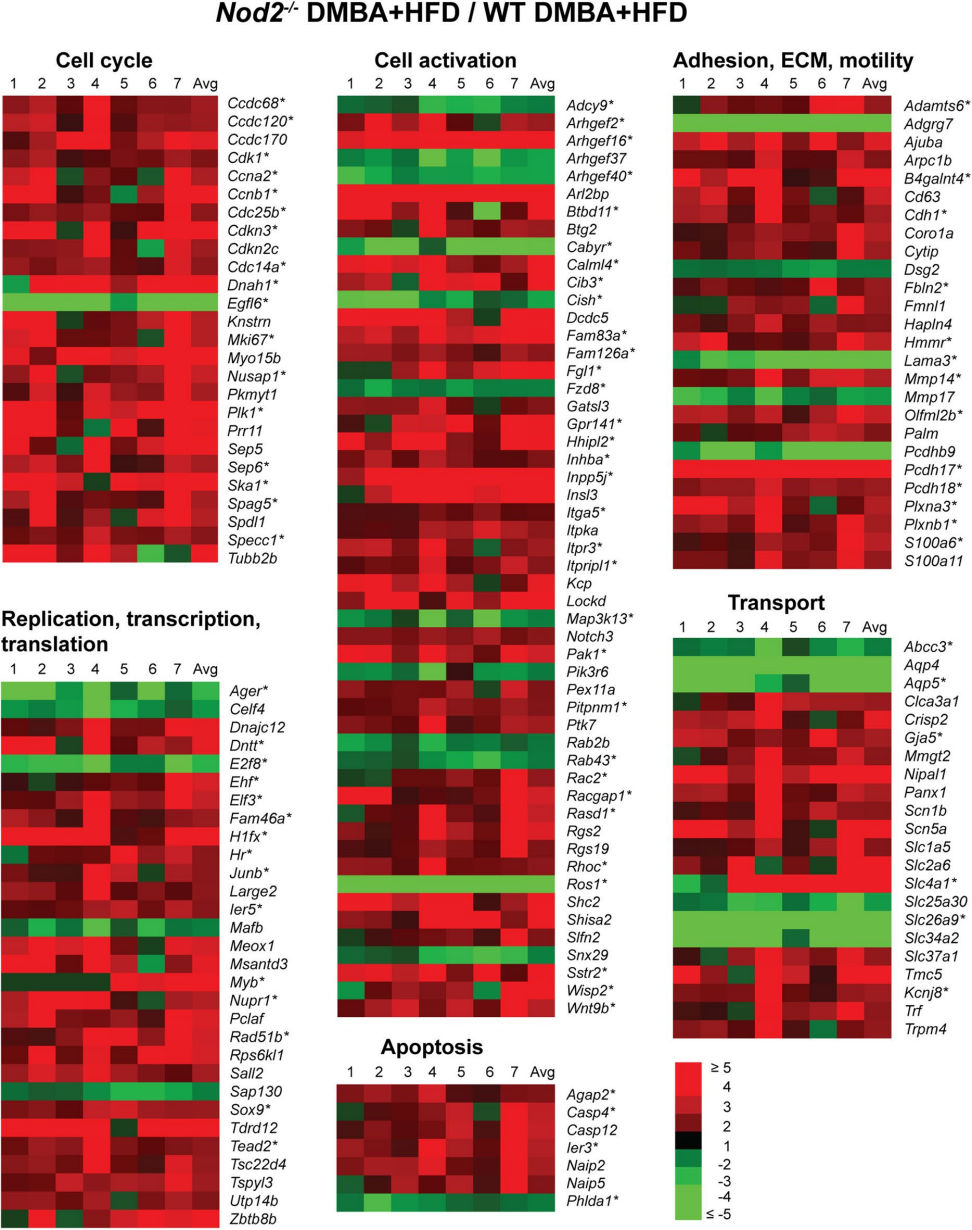
*Nod2*^−/−^ DMBA + HFD mice have increased expression of liver genes that promote cell proliferation. Heatmap representation of the fold increase for gene expression in the liver of *Nod2*^−/−^ mice treated with DMBA and maintained on HFD for 31 weeks compared with similarly treated WT mice. The genes are grouped based on function. The fold ratio for individual *Nod2*^−/−^ mice to the average of WT mice is shown in lanes 1 to 7 and with the average (Avg) of the fold for all *Nod2*^−/−^ mice. Asterisks indicate genes associated with liver malignancy. Genes that have a ≥ twofold increase or a ≥ twofold decrease and *P* ≤ 0.05 with 5% FDR are included in the heatmap. *N* = 6–7 mice/group. The numerical data for RNA levels in individual WT and *Nod2*^−/−^ mice and the fold increase in individual and the average for all *Nod2*^−/−^ mice are shown in Supplementary Table S1.

We identified individual genes that were differentially expressed in the liver of WT and *Nod2*^−/−^ mice treated with DMBA and maintained on HFD for 31 weeks. Of all the genes with significantly changed expression, 379 genes had a ≥ twofold increase and 107 genes had a ≥ twofold decrease; and we focused on these genes. *Nod2*^−/−^ DMBA + HFD male mice had significantly altered expression of 162 genes that are associated with cell proliferation, activation and adhesion, including regulators of cell cycle (*Cdk1*, *Ccna2*, *Ccnb1*), organization of the mitotic spindle (*Knstrn*, *Nusap1*, *Plk1*), activation of cells (*Arhgef2*, *Arhgef16*, *Arhgef37*, *Rac2*) extracellular matrix remodeling, migration, and adhesion (*Cdh1*, *Lama3*, *Mmp14*, *Mmp25*), and apoptosis (*Casp4*, *Casp12*, *Naip2*, *Naip5*) (Fig. 2 and Supplementary Table S1). The majority of these genes are upregulated and promote cell proliferation, cell division, and migration. Using the IPA software, we identified that more than 70 of these upregulated genes are associated with the development and progression of hepatic cancer or more specifically with hepatocellular carcinoma (Fig. 2, marked with an asterisk).

**Figure 3:**
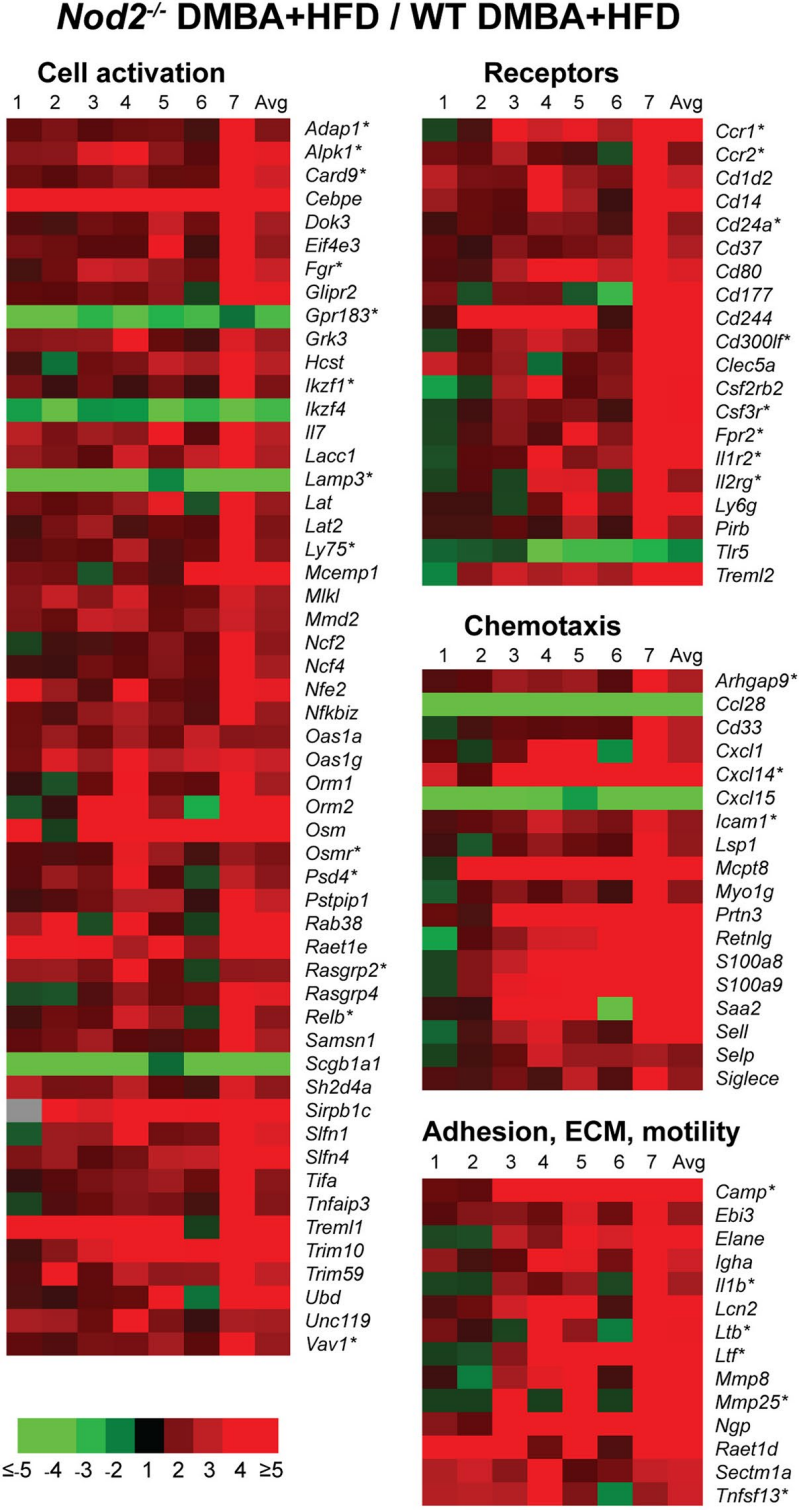
*Nod2*^−/−^ DMBA + HFD mice have increased expression of liver genes for immune responses. Heatmap representation of the fold increase for gene expression in the liver of *Nod2*^−/−^ mice treated with DMBA and maintained on HFD for 31 weeks compared with similarly treated WT mice. The genes are grouped based on function or pathway. The fold ratio for individual *Nod2*^−/−^ mice to the average of WT mice is shown in lanes 1 to 7 and with the average (Avg) of the fold for all *Nod2*^−/−^ mice. Asterisks indicate genes also associated with liver malignancy. Genes that have a ≥ twofold increase or a ≥ twofold decrease and *P* ≤ 0.05 with 5% FDR are included in the heatmap. *N* = 6–7 mice/group. The numerical data for RNA levels in individual WT and *Nod2*^−/−^ mice and the fold increase in individual and the average for all *Nod2*^−/−^ mice are shown in Supplementary Table S2.

*Nod2*^−/−^ DMBA + HFD mice had significantly different expression of more than 100 genes associated with immune cells and immune responses (Fig. 3 and Supplementary Table S2). The vast majority of upregulated genes are involved in the activation and migration of neutrophils and monocytes or code for effector molecules produced by these cells. These genes include molecules associated with immune cell development and activation (*Fgr1*, *Lacc1*, *Cebpe*, *Csf3r*), effector molecules (*Camp*, *Elane*, *Il1b*, *Lcn2*, *Mmp8*, *Mmp25*), chemotaxis and adhesion (*Cxcl1*, *Cxcl14*, *Icam1*, *S100a8*, *S100a9*, *Saa2*), receptors (*Ccr1*, *Ccr2*, *Cd24a*, *Fpr2*), and ROS production (*Ncf2*, *Ncf4*). Using the IPA software, we identified that about 30 of the upregulated immune genes are also associated with the development and progression of hepatocellular carcinoma (Fig. 3, marked with an asterisk).

**Figure 4:**
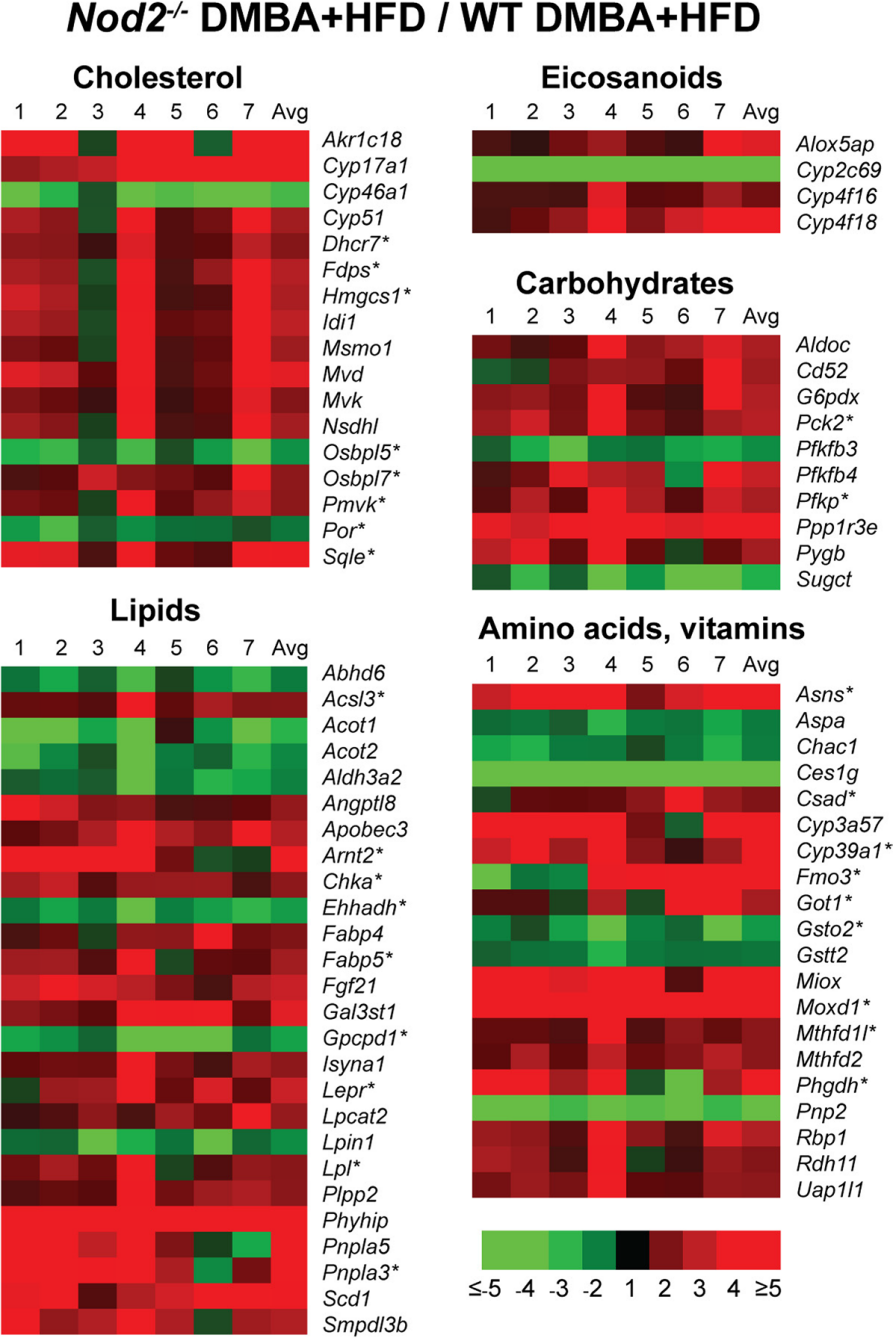
*Nod2*^−/−^ DMBA + HFD mice have increased expression of liver genes for cholesterol biosynthesis. Heatmap representation of the fold increase for gene expression in the liver of *Nod2*^−/−^ mice treated with DMBA and maintained on HFD for 31 weeks compared with similarly treated WT mice. The genes are grouped based on function or metabolic pathway. The fold ratio for individual *Nod2*^−/−^ mice to the average of WT mice is shown in lanes 1 to 7 and with the average (Avg) of the fold for all *Nod2*^−/−^ HFD mice. Asterisks indicate genes also associated with liver malignancy. Genes that have a ≥ twofold increase or a ≥ twofold decrease and *P* ≤ 0.05 with 5% FDR are included in the heatmap. *N* = 6–7 mice/group. The numerical data for RNA levels in individual WT and *Nod2*^−/−^ mice and the fold increase in individual and the average for all *Nod2*^−/−^ mice are shown in Supplementary Table S3.

*Nod2*^−/−^ DMBA + HFD mice had significantly different expression of genes involved in lipid metabolism (Fig. 4 and Supplementary Table S3). In our pathway analysis, we identified significantly enhanced expression of genes in cholesterol biosynthesis (Table 1). Additionally, the top regulators identified by IPA are associated with lipid metabolism. These regulators were cytochrome P450 oxidoreductase (POR, *P* = 1.10E−20), peroxisome proliferator activated receptor alpha (PPARA, *P* = 1.07E−19) and SCAP (*P* = 3.18E−15). POR transfers electrons to all microsomal P450 enzymes, including enzymes in steroid biosynthesis. PPARA, is a transcription factor that regulates the expression of genes in lipid metabolism, cell proliferation and differentiation, and immune responses. SCAP is a protease that cleaves SREBP, a key transcription factor that induces expression of several enzymes involved in cholesterol biosynthesis. Based on the changes in these regulators, *Nod2*^−/−^ DMBA + HFD mice are predicted to have increased cholesterol biosynthesis. We also demonstrate that many genes involved in steroid biosynthesis including *Hmgcs1*, *Idi1*, *Msmo1*, *Mvk*, *Sqle* and in phospholipid biosynthesis, including *Chka*, *Lpcat2*, *Plpp2* were elevated in *Nod2*^−/−^ mice. Increased cholesterol and phospholipids are essential for rapidly dividing cells^27^, and metabolites of cholesterol, including oxysterols, promote tumor development^28^. These results also correlate with our previous results demonstrating hypercholesterolemia in *Nod2*^−/−^ mice on HFD^15^. Genes involved in the biosynthesis of asparagine (*Asns*) and serine (*Phgdh*) and in folate metabolism (*Mthfd1l* and *Mthfd2*) were also elevated in *Nod2*^−/−^ DMBA + HFD mice and are associated with the development of hepatocellular carcinoma. Differentially expressed genes associated with the development of liver cancer based on IPA are marked with an asterisk in Fig. 4.

**Figure 5:**
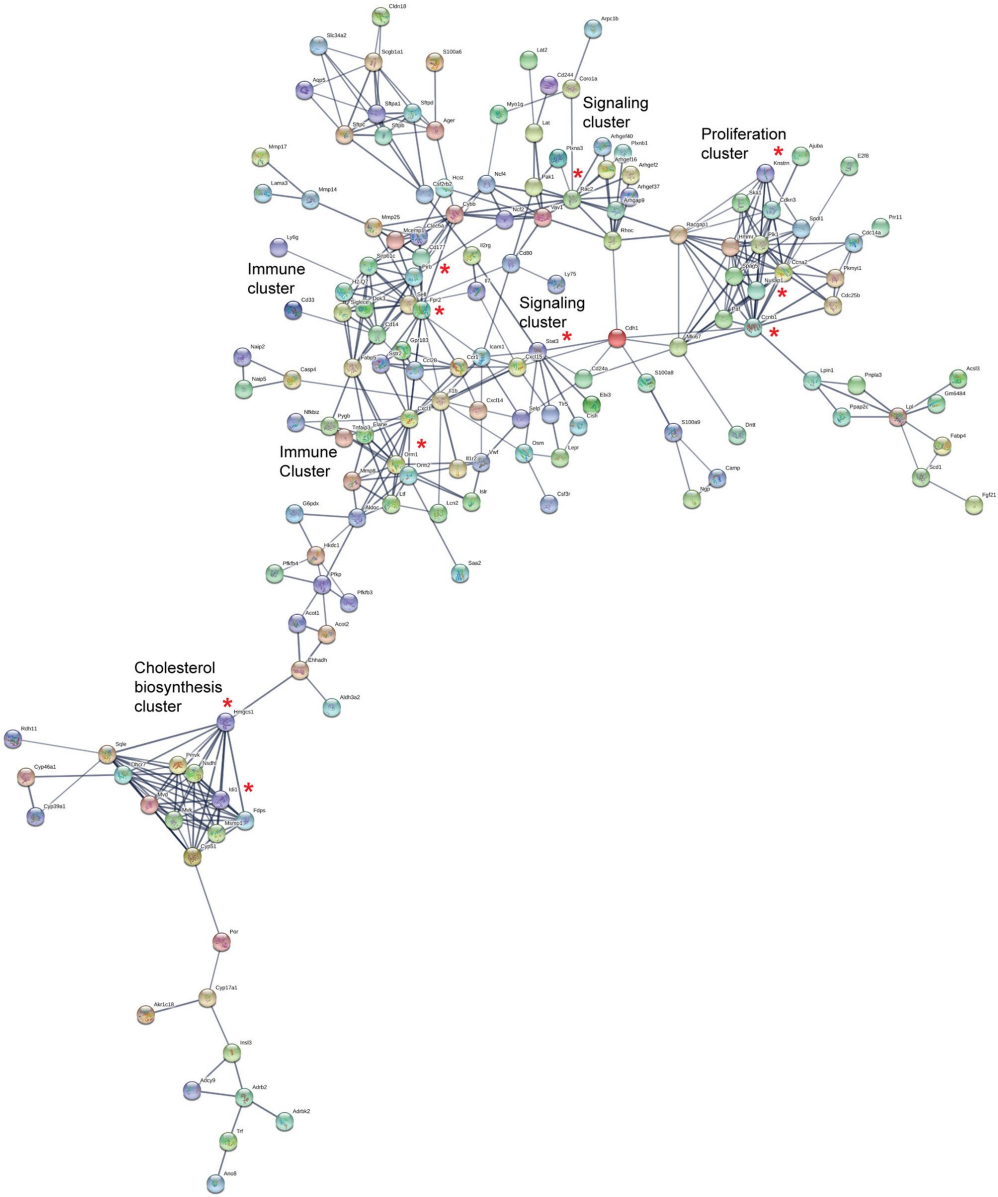
STRING map of predicted protein–protein interactions between differentially expressed genes in *Nod2*^−/−^ DMBA + HFD. Genes that were differentially expressed between WT DMBA + HFD and *Nod2*^−/−^ DMBA + HFD mice were analyzed for protein networks by STRING v11 using the confidence mode. Protein–protein interactions with a combined confidence score of 0.7 or higher are shown and interactions between 5 or fewer proteins are excluded. The interactions between expressed proteins are indicated by the connecting lines and the darker lines represent stronger association compared with the lighter lines. The top ten nodes based on number of interactions are indicated by red asterisk (*) and the top clusters are identified.

We performed STRING analysis to predict protein–protein interactions^29^ for differentially expressed genes in *Nod2*^−/−^ mice treated with DMBA and maintained on HFD for 31 weeks compared with similarly treated WT mice. We performed the analysis with all genes that had a fold change of ≥ 2 and 5% FDR and identified 423 interactions that have a combined score of 0.7 or higher (Supplementary Table S4). A combined score of 0.7 to 0.89 is considered high confidence and 0.9 or higher is considered the highest confidence^29^. We focused on the top ten nodes based on the number of predicted interactions. Based on their function, these proteins could be divided into three major groups: proteins involved in cell proliferation, immune responses and inflammation, and metabolism (Fig. 5, marked with a red asterisk and Supplementary Table S5). We further identified KEGG (Kyoto Encyclopedia of Genes and Genomes) pathways that were significantly enriched (5% FDR, *P* ≤ 0.05) in *Nod2*^−/−^ mice (Supplementary Table S6).

Cyclin B1 (Ccnb1), kinetochore localized astrin binding protein (Knstrn), and nucleolar and spindle associated protein 1 (Nusap1) were the top nodes in the cell proliferation group and formed a tight cluster with several other proteins involved in the regulation of the cell cycle or in the assembly of the mitotic spindle (Fig. 5, marked with a red asterisk and Supplementary Table S5). All the proteins in this cluster have increased expression in tumorigenic *Nod2*^−/−^ mice and likely contribute to the increased cell division and growth (Fig. 2 and Supplementary Table S1). The top nodes in the immune response cluster included formyl peptide receptor (Fpr2), a protein expressed on phagocytic cells that binds formyl peptides, strong neutrophil chemotactic factors; orosomucoid 2 (Orm2), an acute phase response protein; and leukocyte immunoglobulin like receptor B3 (Lilrb3, Pirb), a protein that inhibits activation of leukocytes. These proteins formed two separate but connected clusters with other immune proteins, including, Cd14, Cxcl1, Elane, Ltf, Orm1, and Pirb. The top nodes in the metabolism cluster were enzymes in cholesterol biosynthesis, 3-hydroxy-3-methylglutaryl-CoA synthase 1 (Hmgcs1), and isopentenyl-diphosphate (Idi1), which connected with several other enzymes in cholesterol biosynthesis to form a tight cluster. These proteins have increased expression in *Nod2*^−/−^ DMBA/HFD mice and likely enhance cholesterol biosynthesis (Fig. 4 and Supplementary Table S3).

**Figure 6:**
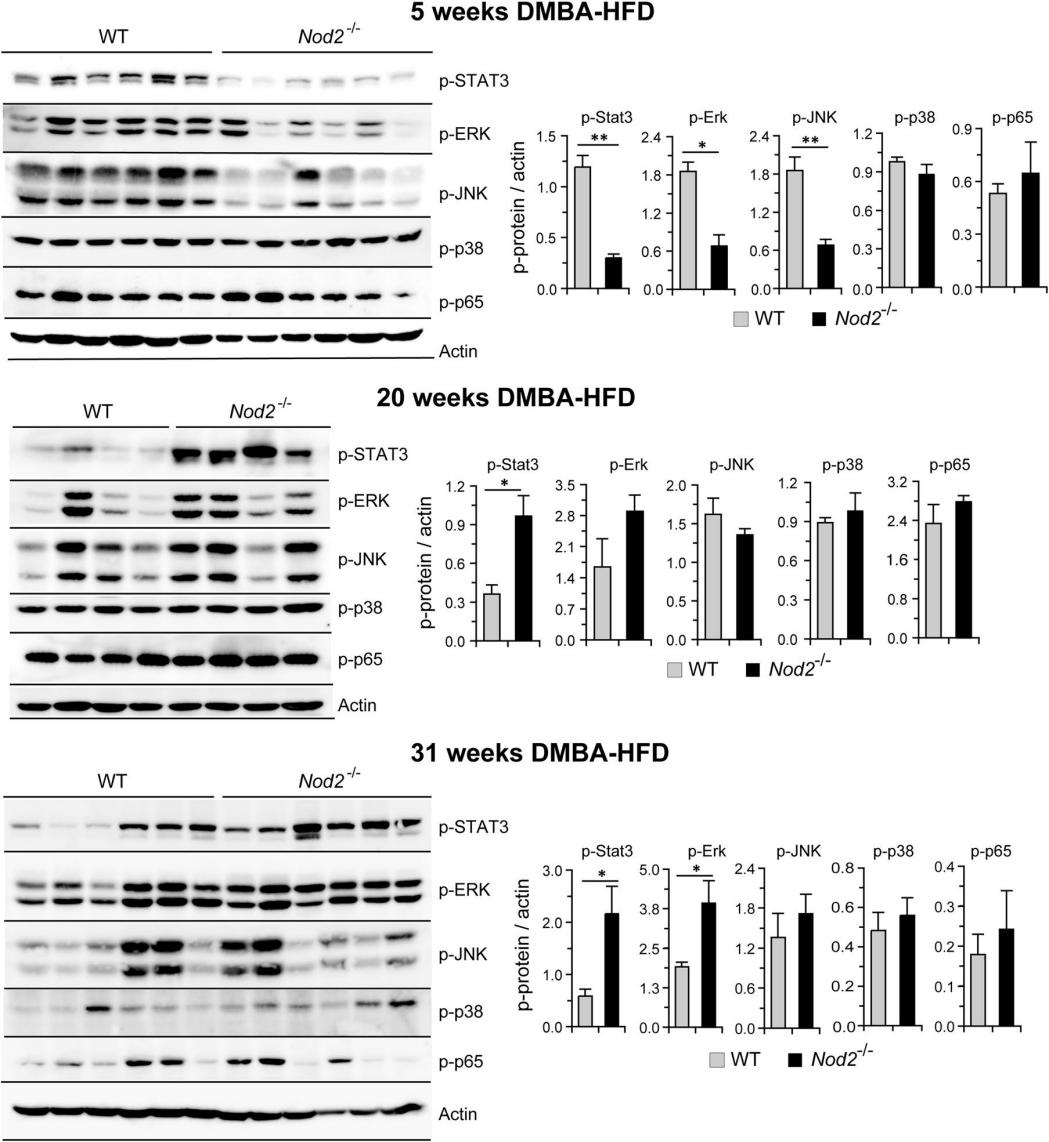
*Nod2*^−/−^ DMBA + HFD mice have increased activation of STAT3 and ERKs. WT and *Nod2*^−/−^ male mice were treated with DMBA and maintained on HFD for 5, 20, or 31 weeks. Liver homogenates were analyzed for activation of signaling molecules using antibodies to p-STAT3, p-ERK, p-JNK, p-p38 and p-p65. Actin was used as a control. Band intensities were measured using ImageJ, ratio of phosphorylated protein to actin was calculated and the fold change in *Nod2*^−/−^ mice over WT mice was determined. The mean fold change ± SEM is graphed. *N* = 4–6 mice/group. Significance of difference by *t-test* for *Nod2*^−/−^ *versus* WT, *P ≤ 0.05, **P ≤ 0.01.

In addition to these three clusters, there were two signaling molecules with high number of interactions, Rac2, a member of the Rac family of GTPases, and Stat3, signal transducer and activator of transcription 3. The Rac2 and Stat3 pathways are activated during cell proliferation and immune responses. The Rac2 cluster included GTPase activating proteins and guanine nucleotide exchange factors. The Stat3 cluster included Cish, a Stat3 inhibitor and Lepr, Il2rg, and Osm proteins involved in Stat3 activation. *Nod2*^−/−^ DMBA/HFD mice have reduced expression of *Cish* and increased expression of *Lepr*, *Il2rg*, and *Osm* (Figs. 2, 3), which likely contribute to the increased activation of Stat3 observed in *Nod2*^−/−^ tumorigenic mice (Fig. 6). The KEGG pathways that were significantly enriched in our STRING analysis included cytokine and chemokine signaling, leukocyte migration, steroid biosynthesis, cell cycle, and Jak-Stat signaling (Table S6).

Thus, our data demonstrate that the increased malignancy in the liver of *Nod2*^−/−^ male mice treated with DMBA and maintained on HFD for 31 weeks, compared with WT mice, is accompanied by changes in gene expression that promote cell division, immune responses, and cholesterol biosynthesis. Our bioinformatic analyses confirm our molecular and IPA results and demonstrate that the pathways enriched in *Nod2*^−/−^ mice are predominantly involved in immune responses, cell proliferation, and steroid biosynthesis. These findings are also consistent with the changes observed during the development of hepatocellular carcinoma in other studies^5–7^.

### Development of obesity-dependent liver tumors in *Nod2*^−/−^ mice is associated with increased activation of the STAT3 and ERK pathways

We next determined the activation of the signaling molecules STAT3, MAPKs (ERK, JNK and p38), and NF-κB, in *Nod2*^−/−^ tumorigenic mice. We selected these signaling pathways because our gene expression and bioinformatic analyses indicate that STAT3 and MAPKs are activated in *Nod2*^−/−^ tumorigenic mice. Furthermore, these three pathways play a critical role in inflammation, proliferation, and the development of many different cancers, and are activated in *Nod2*^−/−^ mice with colorectal cancer^23^. We compared WT and *Nod2*^−/−^ male mice treated with DMBA and maintained on HFD for different times, 5, 20, or 31 weeks, to identify changes in these pathways during the early and late stages of tumorigenesis. We demonstrate that in the liver of *Nod2*^−/−^ mice compared with WT mice phosphorylation (used as a measure of activation) of STAT3 (p-STAT3) was significantly lower at 5 weeks of HFD but significantly higher at 20 and 31 weeks of HFD (Fig. 6). *Nod2*^−/−^ mice had lower level of phosphorylated ERKs (p-ERK) at 5 weeks, no difference at 20 weeks, and higher level of phosphorylated ERK at 31 weeks compared with WT mice (Fig. 6). *Nod2*^−/−^ mice also had lower levels of phosphorylated JNK (p-JNK) at 5 weeks, and there was no difference at 20 or 31 weeks of HFD compared with WT mice. There was no difference in the levels of phosphorylated p38 (p-p38) or NF-κB (p-p65) between WT and *Nod2*^−/−^ mice at any of the time points (Fig. 6). Thus, our data indicate that increased tumorigenesis in *Nod2*^−/−^ mice is associated with decreased activation of STAT3, ERK, and JNK during the early stages but increased activation of STAT3 and ERK during the later stages of tumor development.

### Development of obesity-dependent liver cancer in *Nod2*^−/−^ mice is associated with an increase in immune cells in blood and liver

Our gene expression data demonstrate increased expression of immune-related genes and our bioinformatic analyses predict enhanced immune and inflammatory pathways in the tumorigenic liver of *Nod2*^−/−^ mice. Furthermore, *Nod2* is directly involved in innate immune responses, indirectly regulates adaptive immune responses, and we have previously demonstrated that the development of obesity in *Nod2*^−/−^ mice on HFD is accompanied by a strong immune response in the liver and adipose tissue^15^. Thus, we next determined changes in the number of different immune cells in WT and *Nod2*^−/−^ mice treated with DMBA and maintained on HFD for 5, 20, or 31 weeks or maintained on chow (Ctr). We determined numbers of neutrophils, macrophages and T-cells in the liver, blood, spleen, and bone marrow by flow cytometry. All samples were first gated on CD45^+^ followed by gating for singlets and then viable cells were selected for further analysis (gating strategy is shown in Supplementary Fig. S2).

**Figure 7:**
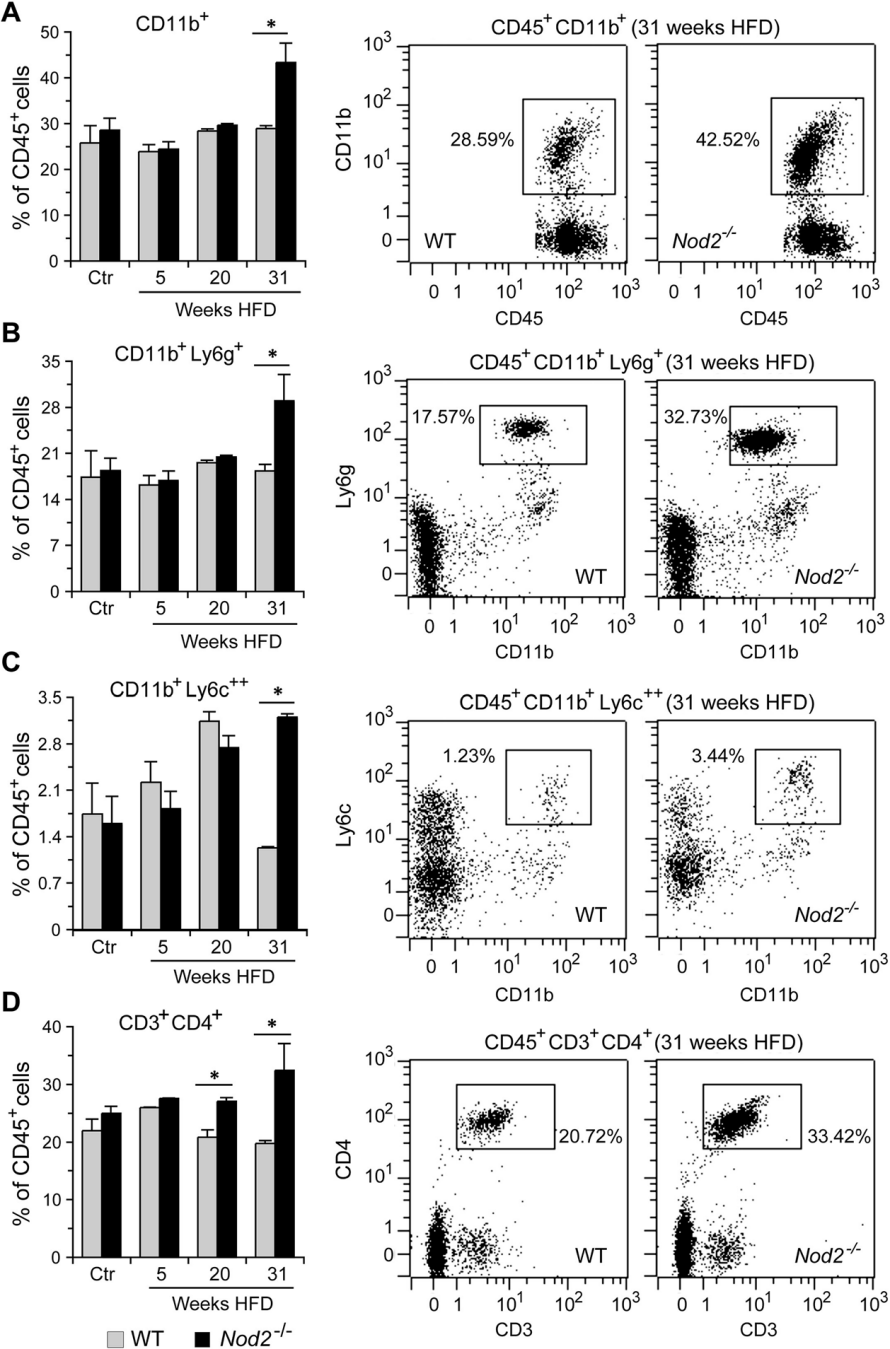
*Nod2*^−/−^ DMBA + HFD mice have increased numbers of neutrophils, inflammatory macrophages, and CD4^+^ T cells in the blood. WT and *Nod2*^−/−^ male mice were treated with DMBA and maintained on HFD for 5, 20, or 31 weeks or on chow for control mice (Ctr). At each time point blood cells were stained with fluorochrome labelled antibodies to CD45, CD11b, Ly6g, Ly6c, CD3, and CD4. Propidium iodide was added before measurement to exclude dead cells. The initial gating strategy included CD45^+^ → singlets → live cells. Viable cells were further gated for (**A**) CD11b^+^, (**B**) CD11b^+^Ly6g^+^, (**C**) CD11b^+^Ly6c^++^, and (**D**) CD3^+^CD4^+^. Mean percent of CD45^+^ cells ± SEM is shown in the left panel and representative dot plots in the right panel. *N* = 5–9 mice/group. Significance of difference was calculated by *t-test* for *Nod2*^−/−^ *versus* WT, *P ≤ 0.05.

In the blood, *Nod2*^−/−^ mice had significantly higher number of CD45^+^CD11b^+^ cells compared with WT mice at 31 weeks of HFD but not at 5 or 20 weeks or in control mice (Fig. 7). CD11b (integrin alpha M, ITGAM) is expressed on the surface of many leukocytes, including monocytes, neutrophils, and dendritic cells, and our results suggest that one or more of these cell populations are increased in the blood of *Nod2*^−/−^ mice treated with DMBA and maintained on HFD for 31 weeks. We also demonstrated that *Nod2*^−/−^ mice treated with DMBA and maintained on HFD for 31 weeks had significantly increased numbers of neutrophils (CD45^+^CD11b^+^Ly6g^+^) and inflammatory monocytes (CD45^+^CD11b^+^Ly6c^++^) compared with similarly treated WT mice (Fig. 7). *Nod2*^−/−^ mice also had significantly increased numbers of CD3^+^CD4^+^ T cells after 20 and 31 weeks of HFD. There was no difference in the numbers of neutrophils, monocytes, or T cells between WT and *Nod2*^−/−^ control mice.

**Figure 8:**
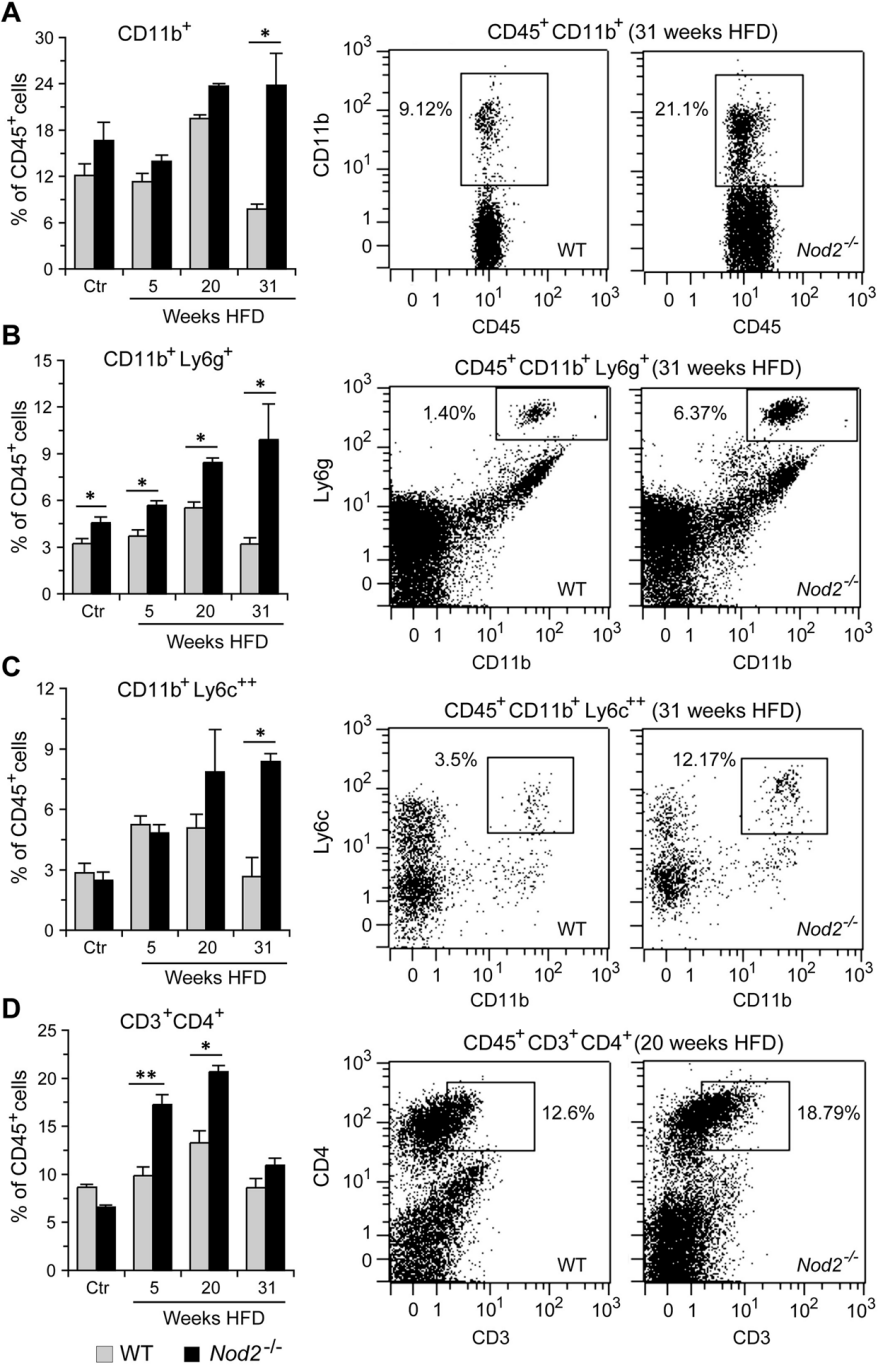
*Nod2*^−/−^ DMBA + HFD mice have increased numbers of neutrophils, inflammatory macrophages, and CD4^+^ T cells in the liver. WT and *Nod2*^−/−^ male mice were treated with DMBA and maintained on HFD for 5, 20, or 31 weeks or on chow for control mice (Ctr). Cells were stained with fluorochrome labelled antibodies to CD45, CD11b, Ly6g, Ly6c, CD3, and CD4. Propidium iodide was added before measurement to exclude dead cells. The initial gating strategy included CD45^+^ → singlets → live cells. Viable cells were further gated for (**A**) CD11b^+^, (**B**) CD11b^+^Ly6g^+^, (**C**) CD11b^+^Ly6c^++^, and (**D**) CD3^+^CD4^+^. Mean percent of CD45^+^ cells ± SEM is shown in the left panel and representative dot plots in the right panel. *N* = 5–9 mice/group. Significance of difference was calculated by *t-test* for *Nod2*^−/−^ versus WT, *P ≤ 0.05, **P ≤ 0.01.

In the liver, *Nod2*^−/−^ mice had significantly higher numbers of CD45^+^CD11b^+^ cells compared with WT mice at 31 weeks of HFD but not at 5 or 20 weeks or in control mice (Fig. 8). Neutrophils (CD45^+^CD11b^+^Ly6g^+^) were significantly higher in *Nod2*^−/−^ mice at all time points as well as in the control mice. However, the total number of neutrophils and the fold increase compared with WT mice for the same group was higher at 31 weeks than at the other time points or in the control mice (control = 1.4, 5 weeks = 1.5, 20 weeks = 1.5, and 31 weeks = 3.1). Inflammatory monocytes (CD45^+^CD11b^+^Ly6c^++^) were significantly higher in the liver of *Nod2*^−/−^ mice at 31 weeks but not at 5 or 20 weeks or in the control mice compared with similarly treated WT mice (Fig. 8). In contrast to neutrophils and monocytes, CD3^+^CD4^+^ T cells increased early and were significantly higher in the liver of *Nod2*^−/−^ mice at 5 and 20 weeks but not at 31 weeks or in the control mice (Fig. 8).

In the spleen, *Nod2*^−/−^ mice had significantly higher numbers of neutrophils at 31 weeks of HFD and CD4^+^ T cells at both 20 and 31 weeks of HFD compared with WT mice (Supplementary Fig. S3A). In the bone marrow, *Nod2*^−/−^ mice had a small but significantly higher number of CD45^+^CD11b^+^ cells and neutrophils at 31 weeks of HFD compared with WT mice (Supplementary Fig. S3B). There was no difference in the numbers of splenic and bone marrow neutrophils or monocytes, or splenic T cells between WT and *Nod2*^−/−^ control mice (Ctr).

We next determined changes in the expression of several immune related genes at different time points. WT and *Nod2*^−/−^ mice were treated with DMBA and maintained on HFD for 5 or 20 weeks or maintained on chow without DMBA treatment, and liver RNA was analyzed by qRT-PCR. *Nod2*^−/−^ mice had significantly increased expression of proinflammatory genes *Ccr1*, *Mmp9*, and *Osm* starting at 5 (Supplementary Fig. S4) and continuing at 31 weeks (Fig. 3 and Supplementary Table S2) of HFD. Ccr1 is a chemokine receptor on monocytes and neutrophils and likely contributes to the increased numbers of these cells in the *Nod2*^−/−^ tumorigenic mice (Figs. 7, 8). Osm is a cytokine in the IL6 family and an activator of STAT3 and may contribute to its increased activation in *Nod2*^−/−^ mice (Fig. 6). *Nod2*^−/−^ mice had significantly increased expression of *Il1b* at 20 weeks (Supplementary Fig. S4) and at 31 weeks (Fig. 3 and Table S2) but not at 5 weeks of HFD. IL1β is a cytokine produced by activated macrophages and increased expression of *Il1b* likely contributes to increased inflammation and cell proliferation in *Nod2*^−/−^ tumorigenic mice. Increased expression of the cytokine *Il6* is often observed in many different tumors, however, there was no difference in the expression of *Il6* at any time point in tumorigenic *Nod2*^−/−^ mice. *Ngp* (neutrophil granule protein), a marker for neutrophils, was significantly increased at 20 weeks (Supplementary Fig. 4) and at 31 weeks (Fig. 3 and Supplementary Table S2) and correlates with the increase in neutrophils in *Nod2*^−/−^ mice (Figs. 7, 8). *Il1r2* codes for a decoy receptor that inhibits the activity of Il1α and Il1β and was significantly increased at the earlier time point but not at 20 weeks (Supplementary Fig. 4). These results further correlate with the decreased activation of signaling molecules at earlier, but enhanced at later time points in *Nod2*^−/−^ mice (Fig. 6).

Altogether, these data demonstrate that the development of HFD-dependent liver tumors in *Nod2*^−/−^ mice is associated with an early lower, but later elevated immune response. The elevated immune response included increased numbers of neutrophils, monocytes, and CD3^+^CD4^+^ T cells in the tumorigenic liver.

## Discussion

We and others have previously demonstrated that the bacterial sensor and innate immunity molecule *Nod2* protects from diet-induced obesity, steatosis, and low-level inflammation^14,15^. These pathologies are risk factors for hepatocellular carcinoma, however, the role of *Nod2* in obesity-associated liver malignancy was not known. In this study, we have identified a novel role for *Nod2* and demonstrate that *Nod2* protects mice from obesity-associated hepatic carcinogenesis. Our results further characterize the molecular mechanisms of *Nod2*-dependent protection from tumorigenesis.

Here we demonstrate that *Nod2*^−/−^ mice treated with the carcinogen DMBA and maintained on HFD for 31 weeks gain more weight and develop more hepatic tumors than similarly treated WT mice. Both, the number and size of tumors in *Nod2*^−/−^ mice were significantly increased. The development of tumors in *Nod2*^−/−^ mice requires both HFD and a carcinogen, as mice maintained on HFD but not treated with a carcinogen and mice treated with carcinogen but maintained on chow did not develop tumors. Furthermore, gender plays a role in susceptibility to malignancy; male *Nod2*^−/−^ mice were significantly more sensitive than female *Nod2*^−/−^ mice to the DMBA + HFD-induced hepatic tumors, however, both males and females are susceptible to diet-dependent obesity compared with WT male and female mice respectively. These results correlate with the gender differences observed in humans; double the number of men die from liver cancer compared with women (American Cancer Society), however, the mechanism behind this difference is not understood.

We demonstrate that *Nod2*^−/−^ mice treated with DMBA and maintained on HFD for 31 weeks had altered expression of genes in pathways associated with the development of cancer, liver hyperplasia, liver carcinoma, liver steatosis, hepatocellular carcinoma, inflammation, and cholesterol biosynthesis. We analyzed the gene expression data for changes in GO pathways and identified increased expression of genes involved in cell division, immune responses, and lipid metabolism. Similar molecular changes have been identified as core transcriptional hallmarks of hepatocellular carcinoma^7^. Our STRING analysis confirmed an enrichment of pathways involved in immune responses, cell cycle, cholesterol biosynthesis, and carcinogenesis in tumorigenic liver of *Nod2*^−/−^ mice, and we also predicted specific protein–protein interactions in these pathways. We further demonstrate that during the later stages of tumor development in *Nod2*^−/−^ mice, STAT3 and ERK are activated, and there are increased numbers of neutrophils, inflammatory monocytes, and CD4^+^ T cells in the blood and liver. However, during the earlier stages there is decreased activation of STAT3 and MAPK in *Nod2*^−/−^ mice compared with WT mice. These results suggest that during the early stages of tumorigenesis *Nod2* is required for the activation of these signaling pathways, however, at later stages of tumorigenesis *Nod2* is required for suppressing the activation of these pathways. These changes may be direct or indirect effects of *Nod2*. Our data further suggest that before tumors appear *Nod2* is primarily responsible for the changes in the signaling pathways and differences in immune cell infiltration, and once tumors appear, both *Nod2* and tumors could be responsible for the modulation of these responses.

Altogether, our results demonstrate that *Nod2* protects from liver malignancy by maintaining optimal levels of cell growth and division, immune responses, and lipid metabolism, and that in the absence of *Nod2* and exposure to a carcinogen, combined with long-term HFD, many of these critical pathways are enhanced and *Nod2*^−/−^ mice become susceptible to liver tumorigenesis.

The relevance of *Nod2* to inflammatory diseases, primarily inflammatory bowel disease (IBD), was demonstrated almost two decades ago^17,18^ and have been followed by numerous studies^11,12^, but the mechanism of the effect of *Nod2* on colitis remains obscure. Polymorphisms in *Nod2* are also linked to colorectal cancer^19–21^, and in experimental models, *Nod2*-deficiency was shown to enhance sensitivity to colorectal cancer with hyperinflammation in the gut^22,23^. Additionally, we and others have demonstrated a novel role for *Nod2* in metabolic dysfunction. In experimental models, *Nod2* deficiency is associated with increased sensitivity to diet-dependent obesity, steatosis, and hepatic and adipose tissue inflammation^14,15^. These results further demonstrate that *Nod2* deficiency combined with different kinds of stress (infection or high fat diet) results in increased inflammation and other diseases including obesity and cancer. However, the mechanism of *Nod2*-mediated pathology remains poorly understood and controversial. Activation of Nod2 results in increased production of inflammatory molecules and anti-microbial peptides^8,9^. The *Nod2* mutations associated with IBD are mainly missense^17,18,30^, which would suggest decreased inflammation in these patients. However, IBD patients have increased inflammation in the colon^31^.

One proposed explanation for increased inflammation associated with *Nod2* deficiency is that Nod2 suppresses signaling through TLRs, which are major contributors to inflammation. In the absence of *Nod2*, TLR activation is unchecked, which results in hyperinflammation. This hypothesis is supported by several studies, which demonstrate that Nod2 inhibits TLR-mediated activation of immune cells and cytokine production^32–34^ and increased intestinal permeability^35^. A recent study demonstrates that *Nod2*, in a colorectal cancer model, induces activation of NF-κB and MAPKs during the early stages of tumorigenesis, yet suppresses TLR-mediated activation of these signaling molecules during the later stages^23^. Our previous and current results demonstrate that the development of obesity and diet-dependent hepatic malignancy in *Nod2*^−/−^ mice is accompanied by increased expression of many immune response genes in the liver and adipose tissue. Here, we demonstrate that *Nod2*^−/−^ mice with hepatic tumors have enhanced molecular and cellular signals in inflammatory pathways. These mice have increased expression of many genes involved in the immune responses in the liver, increased numbers of neutrophils, monocytes, and CD4^+^ T cells in the liver and blood, and increased activation of STAT3 and ERKs in the liver. Thus, one likely explanation for our results is that the development of hyperinflammation in *Nod2*^−/−^ mice maintained on HFD or treated with DMBA and HFD is due to the loss of Nod2-mediated suppression of inflammation, possibly triggered by TLR signaling.

A second parallel mechanism that may contribute to the development of diet-dependent obesity and hepatic malignancy in *Nod2*^−/−^ mice may be the recently described Nod2-mediated activation of AMPK^24^. In that study, the authors demonstrate a novel role for Nod2 in protecting from *N*-nitrosodiethylamine/carbon tetrachloride-induced hepatocarcinogenesis^24^. Using hepatic tumor cell lines, they further demonstrated that Nod2 binds to and activates AMPK signaling, which inhibits mTORC1 resulting in an anti-tumor effect^24^. However, these in vitro results have not been confirmed in vivo. AMPK is a master regulator of metabolism, regulates cell proliferation, and activation of AMPK in the liver reduces liver steatosis and inflammation^36,37^. Thus, one likely explanation for our data demonstrating the development of HFD-dependent hyperlipidemia, steatosis, and inflammation in *Nod2*^−/−^ mice could be due the loss of Nod2-mediated activation of AMPK. Currently, our data only indirectly support these hypotheses and will be confirmed in future.

Nod2 has multiple direct and indirect effects in the body, including modulation of the immune systems, metabolism, and the gut microbiota, and it is likely that multiple mechanisms influence the development of liver tumorigenesis in our current model. In our previous work, we have demonstrated that *Nod2*^−/−^ mice are more susceptible than WT mice to diet-dependent obesity and metabolic disease, and that this susceptibility was in part due the *Nod2*^−/−^ HFD microbiome (15). Thus, it is possible that both the microbiota and metabolic disease contribute to the development of hepatic malignancy, which we will investigate in the future. However, all the observed changes in tumorigenic *Nod2*^−/−^ mice are due to the deficiency of only one gene—*Nod2*, but there may be multiple downstream pathways affected. We have taken precautions in breeding and maintaining our mice (described in the Methods section) to avoid random differences in the environment and the microbiome that may influence disease development.

In conclusion, we have demonstrated that the pattern recognition receptor *Nod2* protects from hepatic malignancy by maintaining optimal levels of cell proliferation, immune responses, and steroid metabolism. These results further support the increasing evidence that *Nod2* possess other functions in addition to the immune responses to bacterial peptidoglycan and that *Nod2* is required for maintaining cellular homeostasis in response to different stresses.

## Materials and methods

### Mice, induction of hepatic tumors, and histopathology

*Nod2*^−/−^ mice on BALB/c background were described previously and deletion of the *Nod2* genes was confirmed by PCR analysis of genomic DNA^15,38,39^. The original founder WT BALB/c mice were obtained from Harlan–Sprague–Dawly. All WT and *Nod2*^−/−^ mice were bred and kept under conventional pathogen-free conditions in the same room in our facility to minimize the influence of differences in the environment. For each experiment, mice from several different cages and breeder pairs were used. Some investigators use homozygous knockout and WT littermates from heterozygous breeding pairs to minimize parent-to-parent and cage-to-cage variations and equalize the microbiomes. We do not use this strategy for two reasons: first, this strategy may skew the results to the particular microbiota present only in this breeding pair. Second and most importantly, we noticed that the effect of innate immunity genes on the composition of microbiota is not instantaneous, but takes time, and stabilization of microbiota characteristic of a given knockout may take more than one generation. We consciously did not want to eliminate or minimize the role of microbiota in our experiments, because the effect of Nod2 on microbiota composition could be one of the possible mechanisms responsible for the observed increased incidence of tumors, which will be determined in the future. The BALB/c background of knockout mice and their negative status for all common viral and bacterial pathogens and parasites (including negative PCR stool tests for mouse Norovirus) were confirmed as previously described^38^. All experiments with mice were approved by the Indiana University School of Medicine-Northwest Institutional Animal Care and Use Committee. All animal work was performed in accordance with Animal Research: Reporting of In Vivo Experiments (ARRIVE) guidelines and regulations.

WT and *Nod2*^−/−^ 4–5 day old pups were treated with 50 µl of 0.5% DMBA, which was applied on their backs^26^ and after 3 days, nursing mothers and pups were placed on HFD (Research Diets, fat 60 kcal%, D12492) for the entire length of the experiment. Mice were placed on HFD diet for 5, 20, or 31 weeks. Control mice were treated with DMBA and maintained on chow for the length of the experiment (up to 35 weeks) or were not treated with DMBA and placed on HFD for the length of the experiment. The pups were weaned at 4 weeks of age and female and male mice were kept in separate cages. Mice were weighed every other week. At the time of sacrifice, tumors were measured using Mitutoyo Vernier caliper for height, weight, and length. In cases when the height of the tumor could not be measured the smallest measurement was taken as the height. The formula V = (π.W.H.L)/6 was used to calculate the volume of each tumor. Blood, liver, spleen, and bone marrow was collected for biochemical and molecular assays. Wherever possible tumor samples were collected separately. Liver samples from WT and *Nod2*^−/−^ mice treated with DMBA and maintained on HFD for 31 weeks were fixed in 10% buffered formalin, embedded in paraffin, sectioned, and stained with hematoxylin and eosin^15^.

### RNA extraction, sequencing, qRT-PCR, and data processing

For identification of genes that were differentially expressed between *Nod2*^−/−^ and WT mice treated with DMBA and maintained on HFD for 31 weeks, we analyzed the total RNA population in the liver by RNA sequencing. RNA was isolated from the liver of individual mice using the TRIZOL method (InVitrogen), followed by purification on RNeasy spin columns using the Qiagen RNeasy Mini Kit^15,40^. RNA sequencing and initial analysis were performed at the Center for Genomics and Bioinformatics, Indiana University Bloomington. Reads were adapter trimmed and quality filtered using Trimmomatic ver. 0.33 (http://www.usadellab.org/cms/?page=trimmomatic), with the cutoff threshold for average base quality score set at 20 over a window of 3 bases. Reads shorter than 20 bases post-trimming were excluded. More than 98% of the reads (on average 22.8 million read pairs per library) passed the quality filters. Cleaned reads were mapped to mouse reference genome GRCm38 (gencode M16), using TopHat2 ver. 2.1.1 (https://doi.org/10.1186/gb-2013-14-4-r36). Approximately 94.7% of the total cleaned reads mapped to the reference and more than 95.5% of those mapped uniquely. RNA sequencing data will be deposited in NCBI.

Differential expression with statistical analysis was calculated using the software DESeq2^15,41^. The fold change was computed for *Nod2*^−/−^ DMBA + HFD with respect to WT DMBA + HFD and genes that had a fold change of ≥ 2 (increased and decreased) and *P* ≤ 0.05 and with a 5% FDR were considered as biologically and significantly different between the 2 groups. Gene products identified as differentially expressed were analyzed to identify biological processes that were altered between the two groups of mice.

To identify genes that were differentially expressed between *Nod2*^−/−^ and WT mice at earlier time points (treated with DMBA and maintained on HFD for 5 or 20 weeks) and between control mice (not treated with DMBA and maintained on chow) we performed qRT-PCR. Total RNA was isolated, converted to cDNA using Applied Biosystems™ High Capacity cDNA Reverse Transcription Kit and real-time PCR was performed using Applied Biosystems™ PowerUp™ SYBR™ Green Master Mix and the Applied Biosystems™ QuantStudio 7 Flex Real-Time PCR system^42,43^. All primers used are listed in Table S7.

### Network analysis of differentially expressed genes

Protein–protein interactions amongst the differentially expressed genes were predicted using Search Tool for the Retrieval of Interacting Genes (STRING) software v11.0 (http://string-db.org)^29^ with the combined score set at ≥ 0.7. STRING uses genomic context information text mining, experimental data, and database searches as sources for interaction criteria^29^.

### Western blots

Liver samples were homogenized in RIPA buffer with 1X protease inhibitor cocktail (P8340, Sigma-Aldrich), 1 mM sodium orthovanadate, and 10 mM sodium fluoride. Tissue debris was removed by centrifugation at 20,000×*g* for 45 min, 4 °C. 40 µg of each protein sample was mixed with 4X sample buffer and kept at 95 °C for 5 min. Samples were centrifuged at 3000×*g*, 1 min, at 4 °C. Proteins were separated on a 10% SDS-PAGE gel at 50 V until the samples reached the separating gel and 100 V after that. Protein samples were transferred to 0.45 µm PVDF membrane at 100 V for 1 h. The membrane was blocked with 5% nonfat dry milk in TBST for 1 h at room temperature and incubated with the primary antibody in blocking solution overnight with constant shaking at 4 °C. The membrane was washed with TBST 4 times for 5 min each, incubated with the secondary antibody, washed with TBST 4X for 5 min each and then developed with the SuperSignal™ West Pico Plus Chemiluminescent or SuperSignal™ West Femto Maximum Sensitivity Substrate. The chemiluminescent signal was captured using GE ImageQuant LAS 4000 and band intensities were measured using ImageJ. All original blots and multiple exposures for some blots are shown in Supplementary Fig. S5. All antibodies were from Cell Signaling Technology. All primary antibodies were rabbit monoclonal and were used at 1:1000 dilution: phospho-NF-κB p65 (Ser536, Cat. # 3033), phospho-p44/42 MAPK (Erk1/2) (Thr202/Tyr204, Cat. # 4370), phospho-STAT3 (Tyr705, Cat. # 9145), phospho-p38 MAPK (Thr180/Tyr182, Cat. # 4511), β-Actin (Cat. # 4970). Anti-rabbit IgG, HRP-linked secondary antibody (Cat. # 7074) was used at 1:2000 dilution.

### Flow cytometry for immune cells from the blood, liver, spleen, bone marrow

Mice were sacrificed, blood was collected in EDTA-coated tubes, EDTA was added to a final concentration of 1.5 mg/ml, and 8 µl was used for the flow staining (Miltenyi protocol). The liver was perfused through the inferior vena cava cannulated with a 25 G needle and perfusion medium (Ca^2+^ and Mg^2+^ free HBSS with 0.5 mM EDTA, pH 8.0)^44^. The medium was pumped through until the liver swelled and then followed by an incision to the portal vein to release the fluid from the liver. Perfusion medium was switched to digestion medium (DMEM with 2 mM calcium and 0.8 mg/ml collagenase D) after the liver lost color. After six to seven min the liver was removed and torn into fine pieces with forceps in 10 ml DMEM. Cells were filtered through 100 µm cell strainer and pelleted. Any remaining red blood cells were lysed using 0.2% NaCl and hepatocytes were pelleted with a 50 g spin. Cells in the supernatant, which included the immune cells, were centrifuged at 450 g for 9 min and resuspended in 2 ml DMEM and 1 × 10^6^ cells were used for flow staining^44^. For single cells from the spleen, the spleen was homogenized in 2 ml 1X BioLegend Red Blood Cell lysis buffer through a 70 µm cell strainer. After 5 min at room temperature, lysis was stopped with 5 ml of wash medium (RPMI with 5% FCS) and the cells were pelleted. Cells were resuspended in wash medium and ~ 1 × 10^6^ cells were used for flow staining^42^. For bone marrow cells, femurs and tibia were cut and cells were flushed using cold RPMI with 10% FBS and 2 mM EDTA. Cells were filtered through a 100 µm cell strainer and pelleted. Red blood cells were lysed using 0.2% NaCl, immune cells were pelleted and washed with cold RPMI with 10% FBS and 2 mM EDTA. Cells were resuspended in 1 ml cold HBSS and 1 × 10^6^ cells were used for flow staining^45^.

All samples were stained with CD45-VioBlue (Cat. #130-110-664), CD11b-PE/Vio770 (Cat. # 130-113-246), Ly6g-APC/Vio770 (Cat. # 130-118-949), Ly6c-VioGreen (cat. # 130-111-784), CD3-FITC (Cat. # 130-119-758), and CD4-APC (Cat. # 130-116-526) from Milteny and CD14-PE (Cat. # 130-119-758) from BioLegend for 10 min at 4 °C, and analyzed by flow cytometry using MACSQuant (Miltenyi) cytometer^42,43,46^. Propidium iodide was added to the samples before measuring to exclude dead cells. CD45^+^ cells were gated to exclude debris, followed by gating for singlets and then for viable cells. Live cells were further analyzed for CD11b^+^Ly6g^+^, CD11b^+^Ly6c^++^, and Cd3^+^Cd4^+^ cells (Supplementary Fig. S2).

### Statistical analysis

Differential expression of genes with statistical analysis was calculated using the software DESeq2 and is described in the RNA sequencing section. The significance of differences for other quantitative results are presented as means ± SEM and were determined by the Student’s t-test using Microsoft Excel or by Chi square. The significance of differences in pathway analysis was determined using the Ingenuity Pathway Analysis (Qiagen) or STRING software^29^. The *N* and *P* values are indicated in the Figures and Tables; *P* ≤ 0.05 was considered significant. The heatmaps were generated using Java TreeView and represent individual and mean fold changes in *Nod2*^−/−^ DMBA + HFD mice relative to WT DMBA + HFD mice, after converting < 1 ratios to negative fold difference using the formula: (− 1)/ ratio in the heatmap. *N* = 6–7 mice/group. The numerical data for RNA levels in individual WT and *Nod2*^−/−^ mice and the fold increase in individual and the average for all *Nod2*^−/−^ mice are shown in Supplementary Table S1.

## Supplementary information

Supplementary Table S1.

Supplementary Table S2.

Supplementary Table S3.

Supplementary Table S4.

Supplementary Table S5.

Supplementary Table S6.

Supplementary Table S7.

Supplementary Figures.

## Data Availability

All supporting transcriptomic data are deposited in NCBI, GEO accession number GSE153833.
