# Supplementary material for: Nod2 protects mice from inflammation and obesity-dependent liver cancer
Source: Sci Rep. 2020 Nov 25;10:20519. doi: 10.1038/s41598-020-77463-7 (PMC7688964; doi:10.1038/s41598-020-77463-7)
Supplement: Supplementary file 8 — Supplementary Figures. [file 41598_2020_77463_MOESM8_ESM.pdf]

Nod2 protects mice from inflammation and obesity-dependent liver cancer

Serdar A. Gurses, Sunil Banskar, Cody Stewart, Bill Trimoski, Roman Dziarski, and Dipika Gupta*

Indiana University School of Medicine–Northwest, Gary, IN 46408, USA

Supplementary Figures

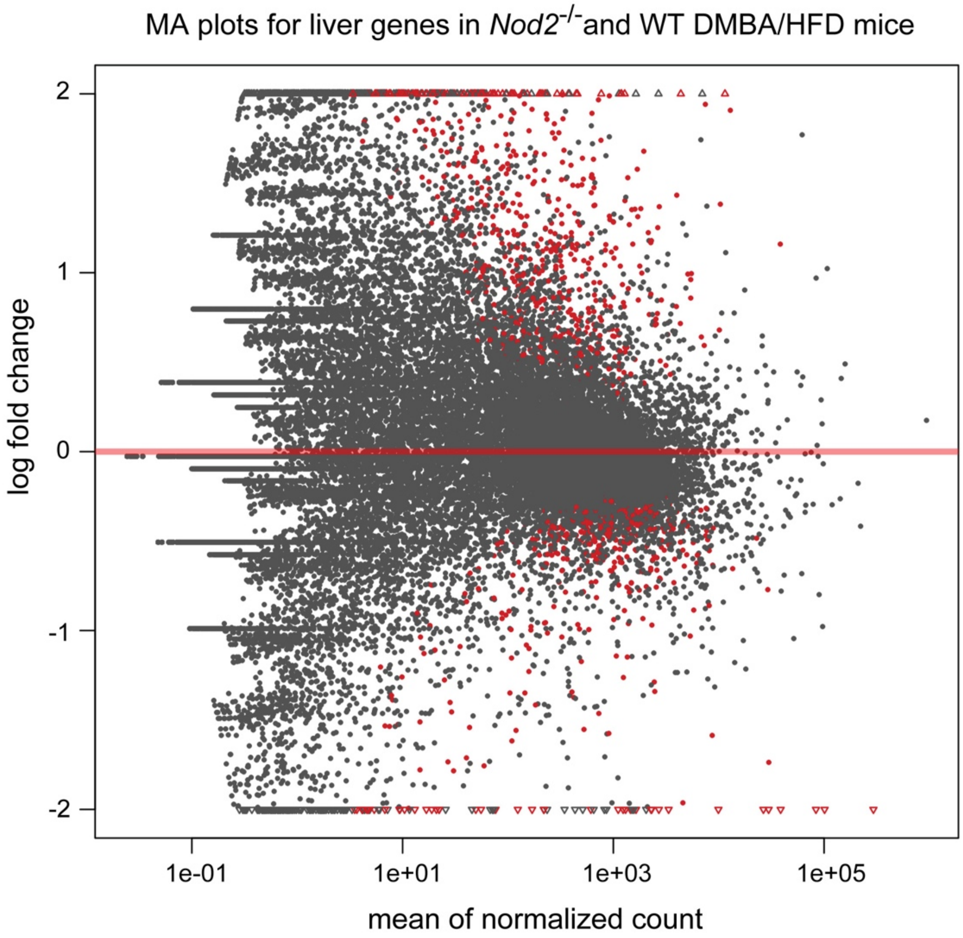

Supplementary Figure S1. _Nod2_$^{-/-}$ DMBA+HFD mice have many differentially expressed genes in the liver compared with WT DMBA+HFD mice. WT and _Nod2_$^{-/-}$ male mice were treated with DMBA, maintained on HFD for 31 weeks and total liver RNA was analyzed by RNAseq. MA plots showing level of gene expression with each dot representing a gene. The Y-axis is the log2 fold change (_Nod2_$^{-/-}$ DMBA+HFD / WT DMBA+HFD) and the X-axis is the average of the counts normalized by size factor. All genes falling on the red horizontal line intersecting at 0 on the Y-axis have a log2 ratio of zero (no change in expression). All genes falling above 0 on the Y-axis are upregulated and all genes falling below 0 on the Y-axis are downregulated. The genes colored red are significantly up or down regulated at P≤0.05 and 5% FDR.

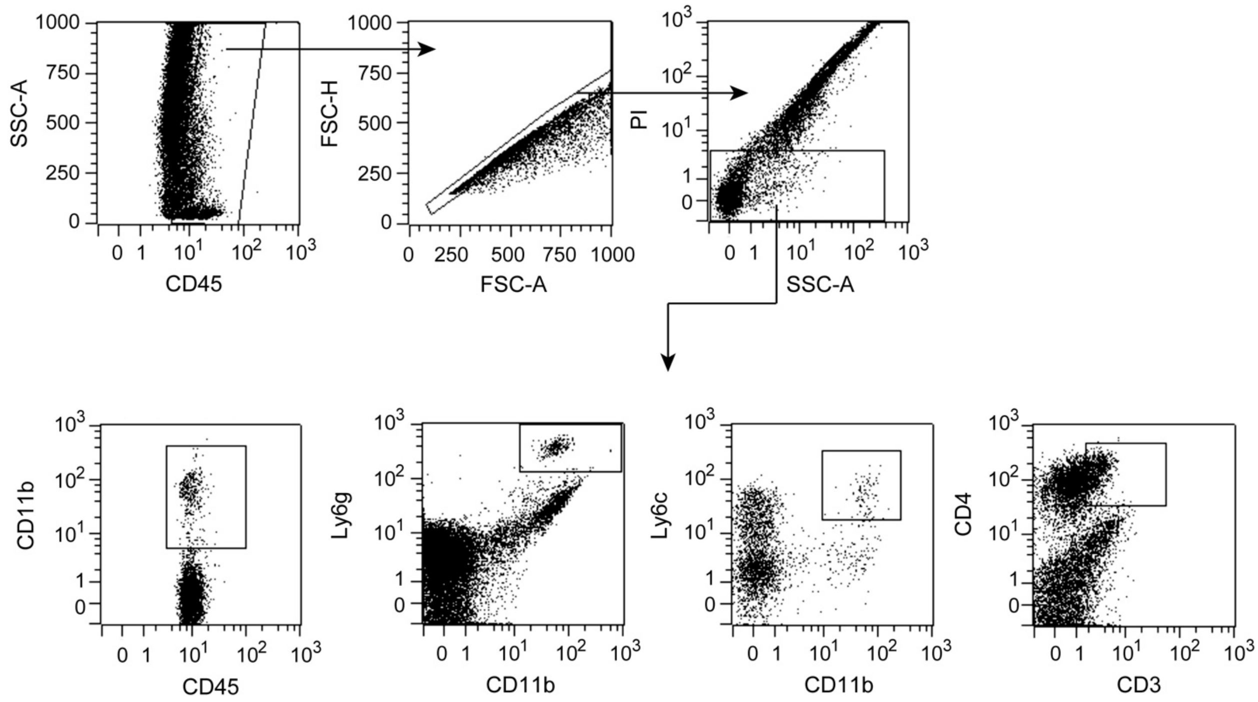

Supplementary Figure S2. Gating strategy for flow cytometry data analysis. Debris was excluded and $CD45^+$ cells selected. Single cells (singlets) were then selected using forward scatter area (FSC-A) versus forward scatter height (FSC-H). Live cells were selected using B2 (Apc-Vio-700-A) versus B3 (PE) channels. Viable cells were further gated for $CD11b^+$, $CD11b^+Ly6g^+$, $CD11b^+Ly6c^{++}$, and $CD3^+CD4^+$. Background fluorescence from unstained cells was used as negative control to define positivity in each channel.

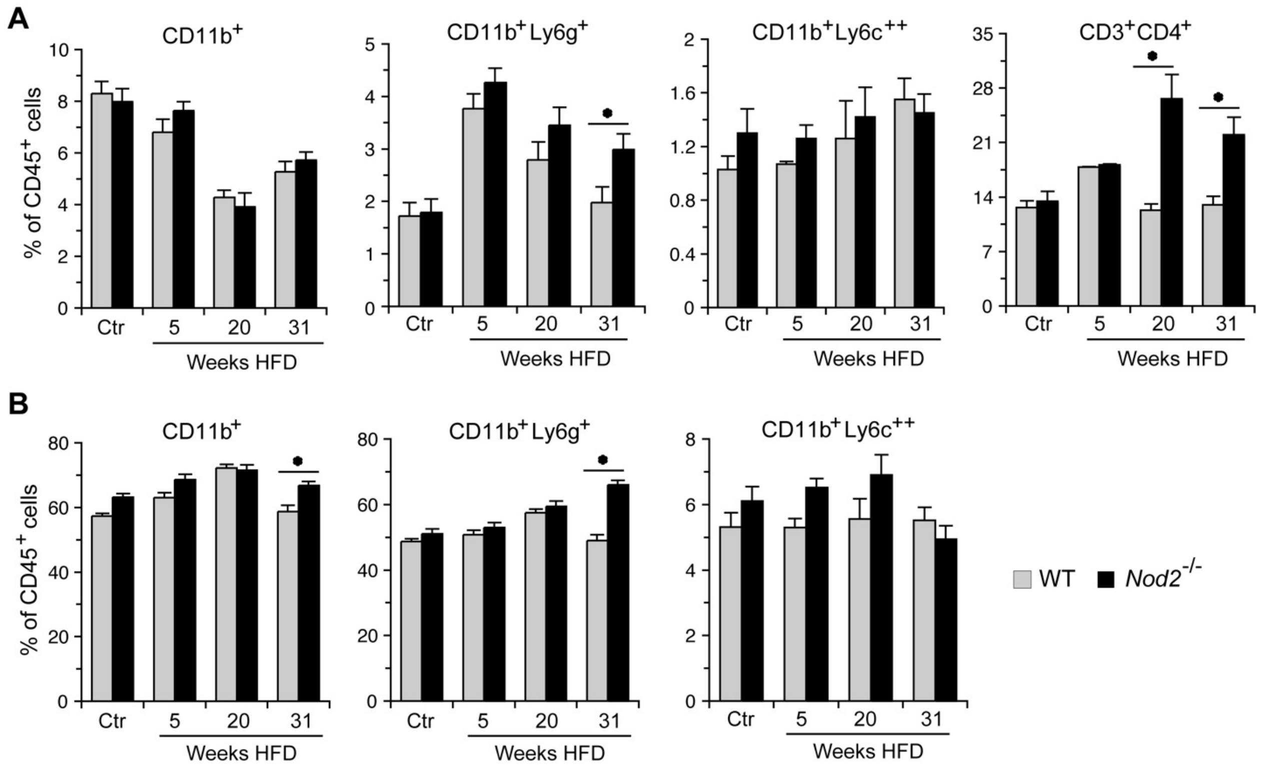

Supplementary Figure S3. Immune cells in spleen and bone marrow of WT and *Nod2*$^{-/-}$ DMBA+HFD mice. WT and *Nod2*$^{-/-}$ male mice were treated with DMBA and maintained on HFD for 5, 20, or 31 weeks or on chow for control mice (Ctr). Cells from (**A**) spleen and (**B**) bone marrow were stained with fluorochrome labelled antibodies to CD45, CD11b, Ly6g, Ly6c, CD3, and CD4. Propidium iodide was added before measurement to exclude dead cells. The initial gating strategy included CD45^{+} → singlets → live cells. These cells were further gated for CD11b^{+}, CD11b^{+}Ly6g^{+}, CD11b^{+}Ly6c^{++}, and CD3^{+}CD4^{+} and mean percent of CD45^{+} cells ± SEM is shown. *N*=4-6 mice/group. Significance of difference by *t-test* for *Nod2*$^{-/-}$ *versus* WT, *$P \leq 0.05$.

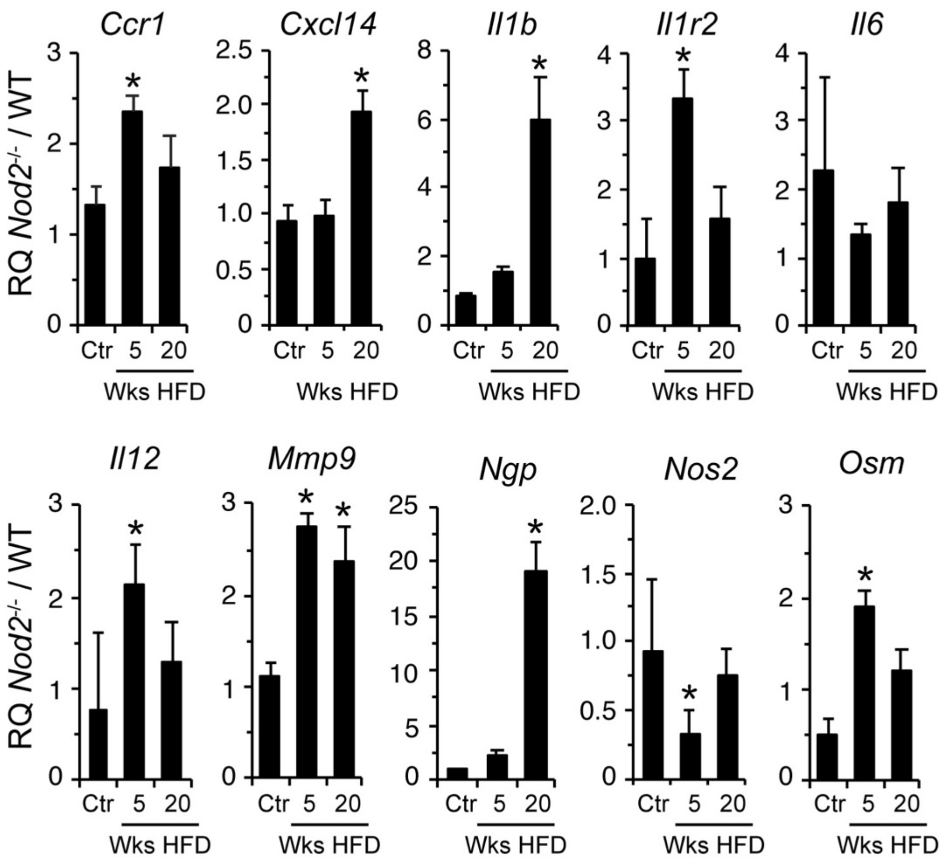

Supplementary Figure S4. ***Nod2***$^{-/-}$ **DMBA+HFD have increased expression of immune genes.** WT and *Nod2*$^{-/-}$ male mice were treated with a single dose of DMBA and maintained on HFD for 5 or 20 weeks and control mice were maintained on chow with no DMBA treatment (Ctr). Total liver RNA was isolated, and gene expression was analyzed by qRT-PCR. The fold change (RQ, Relative Quantity) for *Nod2*$^{-/-}$ DMBA+HFD / WT DMBA+HFD) is graphed. *N*=4-6 mice/group. Significance of difference in expression was calculated using *t-test* with *Nod2*$^{-/-}$ *versus* WT, $^{*}P \leq 0.05$.

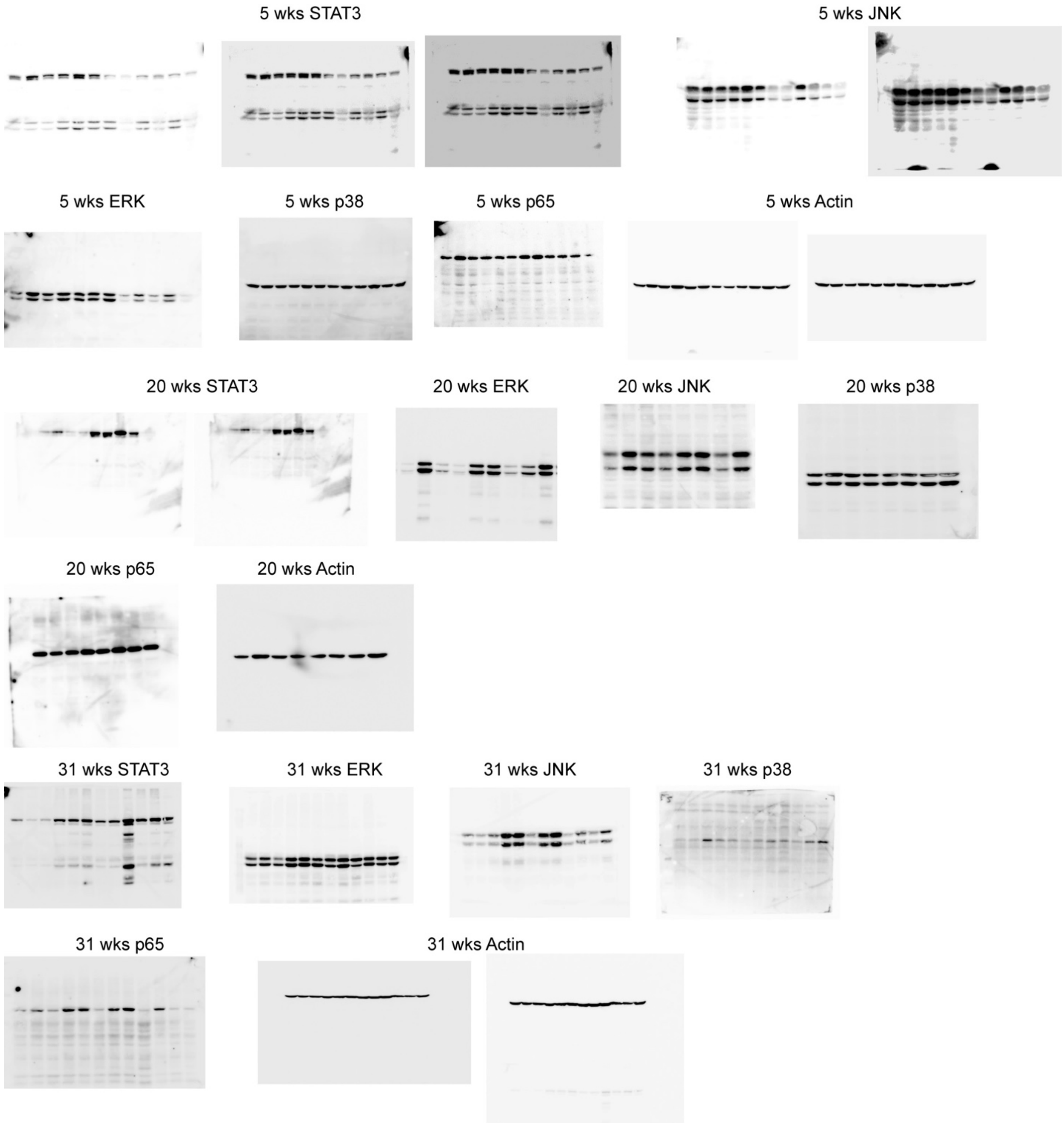

Supplementary Figure S5. Original Western blots. The cropped versions are shown in Figure 6. Some of the blots were stripped and re-probed with other antibodies. For 5 weeks, the p-p38 blot was re-probed for actin. For 20 weeks, the p-p65 blot was re-probed for p-ERK and the p-JNK blot for p-p38 and actin. For 31 weeks, the p-p65 blot was re-probed for actin and the p-ERK blot for p-STAT3. Multiple exposures for 5 weeks STAT3, JNK, and actin, 20 weeks STAT3, and 31 weeks actin are included.
